# Toward a Symptom-Guided Neurostimulation for Gilles de la Tourette Syndrome

**DOI:** 10.3389/fpsyt.2017.00029

**Published:** 2017-02-27

**Authors:** Nicole Pedroarena-Leal, Diane Ruge

**Affiliations:** ^1^Department of Psychology and Neurosciences, Leibniz Research Centre for Working Environment and Human Factors, Technical University Dortmund, Dortmund, Germany

**Keywords:** neuropsychiatry, physiology, symptom, cerebellum, therapy, Gilles de la Tourette syndrome

## Abstract

Therapy resistance of approximately one-third of patients with Gilles de la Tourette syndrome (GTS) requires consideration of alternative therapeutic interventions. This article provides a condensed review of the invasive and non-invasive stimulation techniques that have been applied, to date, for treatment and investigation of GTS. Through this perspective and short review, the article discusses potential novel applications for neurostimulation techniques based on a symptom-guided approach. The concept of considering the physiological basis of specific symptoms when using stimulation techniques will provide a platform for more effective non-pharmacological neuromodulation of symptoms in GTS.

## Introduction

The use of non-invasive and invasive brain stimulation techniques for relief of specific symptomatology in neuropsychiatric disorders should be considered as a young therapeutic intervention. The motivation for such a proposal stems from the need for alternatives to current pharmaceutical neuromodulation for Gilles de la Tourette syndrome (GTS), as approximately one-third of patients with GTS demonstrate therapy resistance or side effects to conventional neuropharmaceuticals, with limited current alternatives for symptom management. When considering the role of circuit components in learning and plasticity processes, brain stimulation becomes a strategic and valuable technique for investigating potential treatment options for neuropsychiatric disorders, since particular neural circuits have demonstrated abnormal excitability related to symptom manifestation and have been linked to aberrations in plasticity-induced learning (1). Such results offer a physiological approach to understanding and analyzing circuit aberrancies that are observed clinically as symptoms of specific neuropsychiatric disorders. The relationship between specific symptom manifestation and the underlying nodal or network participation that accounts for alterations in neural firing is a question worth posing, as such differences in analysis would personalize and thereby improve the current approach to the application and use of neurostimulation.

Of particular interest is the potential to use neurostimulation in a more discrete and motivated manner. Specifically, regions that are analyzed to participate in specific motor and cognitive functions are targeted as regions of interest, an approach guided by the concept of “personalized intervention” in neuropsychiatric disorders. This deviates from the current dominating avenues where a particular structure is chosen for stimulation. Analysis of resulting behavior and physiology, usually at a group level, follows. This paper provides an overview of the application, to date, of both invasive and non-invasive neurostimulation to GTS patients and reflects on the potential benefits and challenges of considering nodal and network participation in aberrant behavior as a potential guide for individualized patient stimulation.

## Function, Structure, and Symptomatology for Motivated Application

The consideration of structures’ functions in the underling neural circuit producing symptomatology is not trivial: stimulation *via* electric currents to a complex electrochemical, dynamic structure that the brain represents prevents simple prediction of potential neurostimulation effects on behavior and symptoms. In essence, this process involves at least three steps: (i) the analysis of the effect of neurostimulation on physiology and (ii) the effect of changes in neurophysiology on behavior or symptoms. These last two steps are preceded by one challenging, cardinal step (A), which in the era of computational neuroscience is often discounted or overlooked: the analysis of the behavioral problem by an experienced pattern recognizer, a clinician. However, some approaches are currently on the way in an attempt to replace or improve human pattern recognition.

So far, in therapeutic neurostimulation for GTS, solid mechanistic rationale for its use is often spared, one reason being that applicators are rarely trained in the previously mentioned aspects, which include (a) clinical pattern recognition, (b) neurophysiology read-outs, and (c) sophisticated stimulation strategies. This delays the development of more effective stimulation protocols. Thus, the need for unconventional options to available treatment can perhaps be best understood by reorienting the manner in which GTS symptomatology is analyzed and framed.

The cardinal behavioral symptoms in GTS are tics. Tics can be sudden and meaningless movements, simple movements (such as blinking, eye rolling, and head nodding) or complex (touching, jumping, squatting, etc.). Often motor tics, including eye blinking and neck turning, are only abnormal and different from regular movements due to the intensity of reoccurrence and frequency (2). Many can develop over time into more purposeful, longer duration movements (complex motor tics). Consequently, *a tic is not a tic is not a tic* and, hence, requires initial analysis in step (A). To add to the complexity, a scenario like this would inadequately reduce the abnormal motor behavior to a one-dimensional behavioral problem (tic), which in GTS, with its frequent comorbidities (obsessions, compulsions, etc.), is often a mixture of faulty motor patterns.

For example, in children, throat clearing and other cold-related behavior is often reported by parents as persisting after the cold recedes and observed to adopt a recurrent and frequent inclusion in motor tic repertoire. Such repeated activation of potentially learned motor memories seems to occur with various motor actions that, if not for their frequent repetition or misplaced execution, would otherwise be considered as regular movements (2). It could be hypothesized that these represent “invasive motor memories,” i.e., learned and stored patterns. Clinical observations such as these frame GTS symptomatology in such a way that allows for a more inclusive approach to analyzing underlying causes of symptoms, as it aims to bridge the underlying physiology with the clinical manifestation reflecting neural aberrancies.

Traditionally, neural nodes that have been attributed to GTS symptoms are elements forming part of the cortico-basal ganglia (BG) network. Such models suggest that involuntary movements are related to decreased inhibitory output from the BG. This reduction in output is thought to underlie excessive frontal cortical activity (2–5). More recently, contributing models have been extended to include cerebellar circuits using functional magnetic resonance imaging (6) and animal models (7). The specific timing with a tic-preceding time interval in the cerebellum and primary motor cortex suggests that both structures function as origins of tic movement release. Furthermore, the match in latency of cerebellar and primary motor cortex discharges proposes that tic expression can be considered to represent a “global network dysrhythmia.” By contrast, local field potential discharges have been recorded in the BG outside tic expression (7). Such difference of involvement in tic production suggests that different nodes function in distinct ways and at varying time points, implying tic-generating networks encompass multiple and distributed areas in neural circuits.

Can both, clinical observation with potential underlying contribution of these various, mentioned nodes to circuit involvement exemplifies be considered in combination? The apparent and observable lack of movement extinction (i.e., in the case of tics: the erasure or inhibition of the undesired movement patterns) could be a faulty pattern in negative reinforcement mediated by aberrant BG processes. Yet, it could be speculated that the early manifestation of tics is mediated *via* cerebellar learning abnormalities, such as the deficient, early correction of error signals and cerebellar eye blink, conditioning type of learning. By contrast, the BG are crucial for the persistence or retention of these same aberrant movements that are impeded from full erasure due to deviant punishment and reinforcement learning. Moreover, the role of the cerebellum and its interaction with anterior nodes, including the amygdala, hippocampus, and prefrontal cortex for extinction of learned movements, but also emotion, fear, and cognitive patterns, has to be taken into consideration. The involvement of different nodes in distinct characteristics of the manifesting symptoms points to the importance and need of considering inter-individual differences in patient tic repertoire or behavioral problems, as such dissimilarities in manifestations among patients could point to variations in nodal participation. Looking at the individual development of tics over time and based on the knowledge we have on learning mechanisms, it becomes plausible that the source of such varied and diverse symptomatology as that present in GTS patients can not be solely attributed to one structure, rather, should be tied to the involvement of wider neural circuit involvement.

How do these patterns, however, persist long enough to be integrated into motor tic repertoire? The nature of certain tics hints at a more diffuse and interconnected network involved in more complex repertoires of tics. These inquiries point to the multidimensional system that is contributing to symptom manifestation. A multidimensional view allows for the consideration that early manifestation of tics is mediated *via* other nodes, for example, the cerebellum, with cerebellar learning abnormalities manifesting as deficient, early correction of error signals and classical conditioning type of learning, while the BG are crucial for the persistence or retention of aberrant movements that cannot be extinguished because of deviant punishment and reinforcement learning.

Parsing out of specific symptom characteristics and their corresponding possible origin allows for a more precisely guided contribution to analyzing nodal participation and circuit level involvement. Such dissection is crucial when considering the application of stimulation, especially in the often comorbid nature of GTS and associated symptoms. This reflection raises two questions: (i) How would stimulating one component of the circuit imply network level effects in remote areas? and (ii) In what way can effects on aberrant motor behavior be extrapolated? One approach to answering such questions could be to shift the focus of stimulation from specific brain targets to stimulation of particular brain networks.

## Invasive and Non-Invasive Neurostimulation for GTS Treatment

Neurostimulation for GTS has thus far been applied without much mechanistic rationale with regard to the underlying neurophysiology mentioned, the specific contribution of nodes and time points in a neural network, nor has the knowledge gap between neurophysiological effects and behavioral effect—if present—been bridged. The following gives a short overview of the approaches that have been used to ameliorate symptoms. This overview provides insight into the fact that stimulation thus far has been target driven stimulation not fully rooted in clear physiological rationale. This serves as a basis for understanding the perspective of symptom-guided targeted neurostimulation explained in previous sections.

Discrete stimulation of anatomical structures to improve aberrant movements has been examined in a wide array of disorders yet, a personalized consideration of the role these areas play as participating nodes in wider neural circuits, and how these circuits come to manifest certain symptoms over others, has not been explicitly attempted nor analyzed in GTS. If clinical benefits outweigh the possible lack of response to stimulation or side effects, this could be considered acceptable, but not having a deeper understanding of underlying mechanism delays progress in development of efficient treatment and leads to dead ends. A tighter analysis of the connection between specific symptom manifestation and nodal structures that could be stimulated for symptom improvement, therefore, could be more fully explored to ground results in physiology and not just in potential epiphenomena.

Relevance of a discussion of DBS or other neurostimulation for movement disorders other than GTS becomes clearer when considering parallels in regards to certain symptomatology. For example, dystonic muscle contractions cause abnormal posturing of limbs, as can occur in certain GTS motor tics. Additionally, dystonic symptoms can resemble GTS tics in their repetitive and involuntary nature. Certain GTS patients also manifest dystonic tics, with movements that are relatively slow and temporarily persistent actions, such as twisting, pulling, or squeezing movements, resembling movements in dystonia patients. Patients exhibiting typical (i.e., clonic) tics have been found to manifest dystonic tics, a common motor manifestation present in 57% of the cohort studied (8). Potentially, this points to similarities in their contributing physiological cause and motivates approaches to studying the application of respective neurostimulation techniques to GTS patients.

In the case of invasive stimulation specifically for GTS, treatment has been applied for severe cases of the disorder. DBS of various thalamic nuclei, the centromedian–parafascicular (CM–pf) and the internal portion of the ventralis oralis anterior, has been used to treat refractory GTS patients (9). Additionally, there have been further tic reduction surgeries for refractory GTS patients following DBS to the anteromedial and postero-ventrolateral portions of the internal globus pallidus, the anterior limb of the internal capsule, subthalamic nucleus, and nucleus accumbens [for an overview, see also Ref. (10) and more recently (11)].

Although such stimulation results indicate the presence of participating anatomical centers in the manifestation of aberrant movement, there appears to be a lacking amount of analysis in terms of the role these various structures have on the production of such erroneous movement, one of the missing factors previously mentioned as necessary for providing neurophysiological backing for stimulating one anatomical component over the other. This lack of bridging between symptomatology and physiological activity indicates that there should be a growing consideration for the stimulation of different, discrete structures for various movement disorders depending on (a) the symptom being targeted and (b) the known, or hypothesized, physiology of nodes of participation in wider neurocircuits underlying such aberrations.

The aforementioned consideration of DBS application in disorders other than GTS sustains relevance when considering similarities. DBS applied to the ventral intermediate nucleus (Vim) of the thalamus has more or less replaced the use of thalamotomy to treat essential tremor (12). In dystonia, the application of DBS to subcortical structures has been used (13). DBS has also been applied to the ventral anterior internal capsule and subgenual cingulate white matter to treat medically intractable forms of certain neuropsychiatric disorders, such as obsessive–compulsive disorder and depression (14). The amygdala has also been proposed as a participating mediator of various neuropsychiatric disorders including anxiety and depression (15). In terms of its role in GTS symptomatology, projections from the superficial nuclei of the amygdala have been considered as important for tic suppression due to the nuclei’s interactions with the frontal cortex (16). Such a neuromodulatory role in tic suppression would implicate the amygdala as another node in the participating circuitry for GTS.

Apart from invasive techniques such as DBS, other physiologically grounded and non-invasive approaches have permitted a relation between symptom manifestation and underlying neural circuits. Abnormal excitability related to symptom manifestation has been linked to aberrations in plasticity-induced learning using transcranial magnetic stimulation (TMS) (1). The electromagnetic stimulation techniques offer both measurement and potential for modulation of neurophysiology using minimal risk and high tolerability methodology. The use of such non-invasive stimulation techniques has been used to activate Purkinje cell circuits of the cerebellar cortex, potentially inducing inhibition of the disynaptic, dentate–thalamocortical facilitatory connection and production of inhibition of the primary motor cortex (M1) (17–19).

Additionally, other stimulation studies have quantified activity of specific structures, such as the cerebellum, involved in aberrant neural circuits and correlated this activity with the severity of specific symptoms, such as motor tics. Such assessment permits relevant circuit activity to be monitored, facilitating targeted application for potential neuromodulation. Specific paradigms have been developed to monitor and quantify structural activity non-invasively. For example, the use of TMS for cerebellar conditioning in healthy subjects, when implemented 5–7 ms prior to the test stimulus, results in inhibition of motor-evoked potentials (20, 21), a decrease in amplitude referred to as cerebellar brain inhibition (CBI). This type of inhibition is mediated through pathways between the cerebellum and M1.

The CBI protocol is an example of a non-invasive neurostimulation paradigm that allows quantification of structural (cerebellar) activity. The amount of activity assessed by the CBI paradigm might be correlated with tic severity, potentially demonstrating that more selective modulation of certain aberrant pathways can be achieved.

Studies have shown that the application of 1-Hz, low-frequency repetitive TMS (rTMS) over the supplementary motor area (SMA) of the cortex in children with GTS led to amelioration of motor tics, with tic symptoms improving over the 12 weeks of the study duration (22). Such improvement is attributed to normalization of bihemispheric hyperexcitability (23). Clinical applications are supported by TMS studies demonstrating that decreased motor inhibition is present in GTS patients, who show physiological differences from non-GTS patients, with a shorter cortical silent period when using cortical inhibition TMS paradigms (24). Additionally, the use of short interval short intracortical inhibition TMS paradigms on children with GTS shows that cortical inhibition is inversely associated with severity of motor tics (25).

Additionally, low-frequency (1 Hz) rTMS has also been applied to normalize overactive motor cortical regions (specifically, the SMA) (26) and improve GTS symptoms. This is based on imaging studies showing that metabolism is increased in premotor, prefrontal, and motor cortex during tic suppression and tics, indicating increased activity in these regions which implicates that hyperexcitability is tied to tic manifestation (27–29). Yet, it has been shown that higher intensities of stimulation (100% of resting motor threshold) administered over more than 2 days has a more significant, long-lasting, and beneficial effect on tics (30) than that of lower frequencies (31). Subjects with GTS who were treated with this TMS paradigm showed clinical improvement during the first week of rTMS and continued to improve during the second week of treatment. This improvement in symptoms was correlated with a significant increase in resting motor threshold, which remained stable 3 months following the study. Clinical improvement is attributed to normalization of the right hemisphere hyperexcitability present in these patients, potentially indicating a restoration of hemispheric symmetry (30).

However, the use of TMS for clinical application remains limited, in part due to the rather narrow interpretation of how these techniques can be best applied. Current problems with stimulation can perhaps be considered to be rooted in the bottom-up approach to its application rather than the top-down proposal previously mentioned: that is, observing clinical manifestation of behavior in order to propose underlying circuits that have structures accessible for specific neurostimulation paradigms designed to modulate particular aberrant firing. The benefit of such a proposal is that it allows consideration of individual symptoms as guides to the physiology involved in their production.

The application of non-invasive neurostimulation techniques for premeditated, symptom-guided application correlated with known or proposed circuit level participation in aberrant physiology has not been clearly described as the prime motivation behind structure targeting or specific clinical improvements. As a result, although there are improvements in certain symptoms resulting from the application of neurostimulation to various anatomical components participating in the underlying circuits of aberrant movements, it must be noted that without a clearer, symptom-guided application of neurostimulation to participating circuit nodes, such modulation of symptoms to specific paradigms or parameters applied cannot decisively be attributed to the resulting changes.

Gilles de la Tourette syndrome patients have been treated with DBS since 1999, and approximately 48 published studies report some degree of motor tic reduction (32). While initial trials have been promising, the mechanisms subserving the effectiveness of DBS in reducing GTS signs and symptoms have yet to be identified. A condensed list of invasive stimulation is provided in Table 1.

**Table 1:** **Examples of Invasive Stimulation in GTS. The table provides a condensed overview of invasive stimulation approaches presented in this article, as well as additional studies of interest, that have been used thus far to ameliorate or investigate symptoms in GTS**.

Reference	*n*	Target	Outcome
Visser-Vandewalle et al. (33)	3	Thalamus: centromedian nucleus, substantia periventricularis, and nucleus ventro-oralis internus	Tic reduction ranging from 72.2 to 90.1% reduction at long-term follow-up, although specific tic persistence was reported for the 3 patients
Maciunas et al. (34)	5	Thalamus: centromedian–parafascicular (CM–Pf) and ventralis oral complex (Voi)	3 out of 5 patients’ improvement in [Yale Global Tic Severity Scale (YGTSS) and TS Symptom List scores]. 2 patients did not improve after 3-month follow-up
Servello et al. (35)	18	Thalamus: bilateral CM–Pf and Voi	6 patients showed progressive improvement in tic severity, and 12 showed recurrent motor and phonic tics after stimulation, with 3 showing waxing and waning of symptoms
Welter et al. (36)	3	Thalamus: bilateral CM–Pf GPi: ventromedial locations	YGTSS showed that GPi stimulation reduced tic severity to 65, 96, and 74%, respectively. Bilateral stimulation of the CM–Pf produced a 64, 30, and 40%, respectively, reduction in tic severity. In patient 2, the improvement decreased after 2 months
Porta et al. (37)	15	Thalamus: bilateral CM–Pf and Voi	15 out of 18 patients improved in tic severity and behavioral ratings (including anxiety and depression) Data not available for 3 patients
Kwon et al. (23)		Transcranial magnetic stimulation (TMS). Supplementary motor area (SMA) of the cortex	Normalization of the right hemisphere hyperexcitability leading to clinical improvement
Martínez-Fernández et al. (38)	5	GPi: 2 patients in the bilateral posterolateral location, 2 patients in the bilateral anteromedial location, 1 initially in the posterolateral was switched to the anteromedial location after 18 months	2 patients with stimulation the bilateral posterolateral location: 1 patient showed a 54.7% reduction in motor tics and the second patient only showed a plateau in motor and phonic tics but still interfered with lifestyle according to YGTSS 2 patients in stimulation in bilateral anteromedial location: 1 patient showed a 60% reduction in motor tics. Motor and phonic tics resolved for the second patient 1 patient with anteromedial location switch after 18 months: only a 19% reduction for motor tics in the YGTSS
Cannon et al. (39)	11	GPi: bilateral anteromedial globus pallidus internus	10 patients (91%) showed improvement in tic severity, with a 48% reduction in motor tics and a 56.5% reduction in phonic tics. 6 patients (54.5%) had more than 50% reduction; sustained for at least 3 months in YGSS. 2 patients required pharmacotherapy for tics after surgery. 1 patient was a non-responder
Maling et al. (40)	5	Thalamus: bilateral CM–Pf and Voi	3 out of 5 patients showed significant YGTSS decrease. The remaining 2 showed only a small clinical improvement corresponding to small changes in gamma power
Porta et al. (41)	18	Thalamus: bilateral CM–Pf and Voi	Reduction in tic severity, as well as improvements in the comorbid obsessive–compulsive behaviors (OCB), and co-existing psychopathologies (anxiety and depressive symptomatology). However, only 7 out of 15 patients did the overall global assessment of improvement indicate improvement
Huys et al. (42)	8	Thalamus: bilateral for 6 patients; ventral anterior and ventrolateral motor areas	YGTSS showed a 51% reduction in motor tics and a 53% in vocal tics, with a total of 58% reduction score compared to baseline
Dehning et al. (43)	6	GPi: bilateral postero-ventrolateral location	2 patients did not respond to stimulation, and the mean YGTSS score for the remaining 4 patients was 29.5 at the last follow-up, with a mean Tourette Syndrome Quality-of-Life Scale (TSQOL) of 7.75
Zhang et al. (44)	13	GPi: bilateral posterolateral location	YGTSS scores at the last visit compared with baseline were reduced in all 13 patients by a mean of 52.1%. 12 of the 13 showed a mean TSQOL of 45.7%, with 1 patient denying improvement. Only 6 patients reported a significantly high response with overall marked reduction in all tic types
Bloch and Levkovitz (45)	12	TMS. Bilateral SMA inhibition	Improvement in clinical symptoms in children with GTS for at least 6 months
Kefalopoulou et al. (46)	15	GPi: bilateral anteromedial location	Mild improvement in YGTSS, with 80.7 for the off-stimulation period, and 68.3 for the on-stimulation period form a baseline of 87.9. No significant improvement in mean quality-of-life scores (GTS-QOL)

*The table provides a condensed overview of invasive stimulation approaches presented in this article, as well as additional studies of interest, that have been used thus far to ameliorate or investigate symptoms in GTS. Beyond the demonstration of DBS as a therapeutic option, the number of insufficient responders is shown*.

Current models of GTS hypothesize that thalamocortical–BG dysfunction is a key network underlying many of its symptoms. A recent study provided evidence that different types of tics, for example, are paralleled by different types of electrophysiological signatures (47): studied patients with GTS implanted with bilateral DBS electrodes with depth leads in the CM–pF as well as subdural strips over the precentral gyrus. Low-frequency (1–10 Hz) CM–pf activity was observed during tics, as well as modulations in beta rhythms over the motor cortex. The division of tics in the study (three categories: long complex, complex, and simple) showed that long complex tics, tics involving multiple body regions and lasting longer than 5 s were synchronized with detectable thalamocortical signatures. Such symptom-categorized monitoring of neural activity in circuitry implicated in GTS physiology highlights the need for more discrete and motivated application of neuromodulation and monitoring, so as to provide firmly guided evidence for prioritizing stimulation and ideally predicting potential outcomes. Acute trials of closed loop stimulation using the human tic detector are currently underway. Such results further indicate that there is neurophysiological evidence for divergent symptom signatures rooted in particular network-firing aberrancies. A recent study by the same group also modified the stimulation patterns to intermittent stimulation of the thalamus and despite reached responder status in the majority of the small patient group (48). A recent review discusses the relevance of different anatomical structures and modes of stimulation, closed loop, open loop, etc. (49).

## Therapeutic Application of Neurostimulation Techniques Based on Plasticity-Induced Learning

The relevance of such physiological considerations versus epiphenomena of therapeutic neurostimulation can be illustrated by reflecting on the possible symptom improvement outcomes of such a circuit-based model of application, i.e., demonstration of causality relationships of stimulation, effects on physiology (perhaps using non-invasive stimulation), and ultimately effects on behavior.

Considering differences in learning specialization, one can propose that targeting particular nodes based on their involvement in specific tic repertoire might be a more efficient manner of using currently available neurostimulation techniques. The cerebellum is important during early phases of abnormal motor learning based on faulty encoding of errors. BG might point to a more context dependent role in the reinforcement of tics, such as reward-based reinforcement (50). As a result, abnormal reinforcement may facilitate repetitive behaviors and may be involved in higher cognitive symptoms of GTS.

Additionally, it is also crucial to consider neural mechanisms that occur offline, more specifically, a consideration of the process of forgetting such retained movements (tics). When contemplating the effects on symptomatology related to such potential decrease in “forgetting mechanisms” (51), aberrancies in this type of synaptic modulation could be proposed to manifest, or at least retain, the erroneous reinforcement of aberrant movements (tics) in these patients.

Therefore, when contemplating the therapeutic application of neurostimulation for GTS symptomology, it is worth considering that plasticity-induced learning is faulty in GTS in more than one way and might originate symptoms that require stimulation of particular nodes over others. There is no simple extrapolation possible to predict an outcome on behavior with current strategies. The analysis to the use and application of neurostimulation requires an interdisciplinary approach to patient symptoms, one in which there is a bridging between clinical eye and investigative complex methods of studying effects on neurophysiology and behavior.

## Conclusion

The parameters for therapeutic use of neurostimulation in neuropsychiatric disorders, as well as the reliability of stimulating certain nodes over other regions for specific symptoms, have yet to be established. Regardless of these present uncertainties of the benefits of neurostimulation techniques for neuropsychiatric disorders and the exact role of certain structures in GTS symptomatology, it is apparent that promising therapeutic alternatives for patients can be developed by considering the application of brain stimulation to neural circuits. However, such application is dependent on finding its use on the modulation of plasticity mechanisms and alteration of learning at a circuit level. Although the benefits of applying neurostimulation techniques as therapy remain to be precisely defined, it is evident that utilizing non-pharmacological neuromodulation techniques is a consideration worth making.

## Author Contributions

All authors were involved in the study concept and design, analysis and interpretation of manuscript, critical revision of manuscript for intellectual content, literature review, acquisition of data, and drafting of the manuscript.

## Conflict of Interest Statement

The authors declare that the research was conducted in the absence of any commercial or financial relationships that could be construed as a potential conflict of interest. The reviewer JK and handling Editor declared their shared affiliation, and the handling Editor states that the process nevertheless met the standards of a fair and objective review.
